# Study on High-Temperature Constitutive Model and Plasticity of the Novel Cr-Mo-V Hot-Work Die Steel Forging

**DOI:** 10.3390/ma17246071

**Published:** 2024-12-12

**Authors:** Yasha Yuan, Yichou Lin, Wenyan Wang, Bo Zhang, Ruxing Shi, Yudong Zhang, Jingpei Xie, Chuan Wu, Feng Mao

**Affiliations:** 1 School of Materials Science and Engineering, Henan University of Science and Technology, Luoyang 471023, China; 15138753540@163.com (Y.Y.); xiejp@mail.haust.edu.cn (J.X.);; 2 Luo Yang CITIC HIC Casting and Forging Co., Ltd., Luoyang 471039, China; 15937917107@163.com (Y.L.); zhangbo32103@126.com (B.Z.); 15038647370@163.com (R.S.); zlwzyd07121129@163.com (Y.Z.); 3 Longmen Laboratory, Luoyang 471000, China; 4 National-Local Joint Engineering Laboratory of Intelligent Manufacturing Oriented Automobile Die & Mould, Tianjin University of Technology and Education, Tianjin 300222, China; wuchuan@tute.edu.cn

**Keywords:** hot-work die steel, hot compression test, high-temperature constitutive model, 3D hot processing maps, plasticity

## Abstract

In response to the increasingly strict performance requirements of large molds, a novel Cr-Mo-V hot-work die steel has been developed. In order to study the high-temperature hot deformation behavior and plasticity of the novel steel, hot compression tests were conducted on the Gleeble-1500D thermal simulation testing machine at a deformation temperature of 950~1200 °C and a strain rate of 0.001~5 s^−1^. Based on the Arrhenius constitutive model, a novel Cr-Mo-V steel high-temperature constitutive model considering strain was established. The reliability and applicability of this modified model, which includes strain compensation, were assessed using the phase relationship coefficient (R) and the average absolute relative error (AARE). The values of R and AARE for comparing predicted outcomes with experimental data were 0.98902 and 3.21%, respectively, indicating that the model demonstrated high precision and reliability. Based on the Prasad criterion, a 3D hot processing map of the novel Cr-Mo-V steel was established, and the instability zone of the material was determined through the hot processing map: the deformation temperature (950~1050 °C) and strain rate (0.001~0.01 s^−1^) were prone to adiabatic shear and crystal mixing. The suitable processing range was determined based on the hot processing map: The first suitable processing area was the strain range of 0.05~0.35, the temperature range was 1100~1175 °C, and the strain rate was 0.001~0.009 s^−1^. The second suitable processing area was a strain of 0.45~0.65, a temperature of 1100~1200 °C, and a strain rate of 0.0024~0.33 s^−1^. Finally, the forging process of hundred-ton die steel forging was developed by combining 3D hot processing maps with finite element simulation, and the forging trial production of 183 t forging was carried out. The good forging quality indicated that the established hot processing map had a good guiding effect on the production of 100-ton test steel forging.

## 1. Introduction

With the development of the mold industry toward large-scale, complex, precise, high-efficiency, and fast-paced directions, its service environment is becoming increasingly demanding [1,2,3], which puts higher requirements on mold materials. The world’s largest 80,000-ton forging press utilizes a mold material composed of 55NiCrMoV7. However, the increasing application of ultra-high-strength steels in forged components—such as the landing gear for the C919 aircraft and rocket engine casings—has led to a marked reduction in the service life of 55NiCrMoV7. This study investigates an innovative material developed by adjusting the molybdenum (Mo) and vanadium (V) content within the framework of 55NiCrMoV7. Both Mo and V are acknowledged as effective carbide-forming elements that facilitate the precipitation of fine, dispersed MC and M_2_C strengthening phases, thereby enhancing high-temperature properties to satisfy the stringent requirements associated with large mold steel applications under elevated temperature and pressure conditions. Due to the substantial size of 100-ton die steel forgings, combined with the exceptional thermal strength of novel materials, significant resistance to large deformations is encountered. If processing is not meticulously controlled, challenges, such as inadequate refinement of coarse grain structures at critical locations, insufficient bonding in shrinkage cavities or porosity issues, and potential initiation of forging cracks, may arise. Therefore, it is imperative to investigate their high-temperature plasticity characteristics to ascertain optimal forging parameters while also identifying deformation conditions that could precipitate instability phenomena, such as crack formation. The high-temperature stress–strain curve reveals the thermal deformation behavior of materials, providing a basis for formulating forming processes and selecting equipment parameters [4,5]. Mold steel forgings have large specifications and high production costs. Using numerical simulation technology to guide production can help locate process parameters within a more precise range and significantly reduce costs. Accurate high-temperature rheological stress models are an important foundation for numerical simulation analysis [6,7]. Therefore, it is necessary to establish an accurate constitutive model, further study the plasticity of the novel Cr-Mo-V steel, optimize process parameters, and improve the performance of forged products.

In recent years, in order to describe the flow behavior of high-Mo- and high-V-type mold steels, scholars have established various constitutive models, such as the mechanical threshold stress (MTS), Bammann–Chisa–Johnson (BCJ), Johnson–Cook, and Arrhenius models, etc. Physical-based constitutive models, such as the MTS model [8] and BCJ model [9], focus on describing the microscopic mechanisms of rheological processes. However, the complexity of these models limits their applications. Empirical constitutive models, such as Johnson–Cook and Arrhenius models, do not require analysis of the physical essence of plastic deformation, are intuitive and easy-to-define parameters, and are widely used. Johnson and Cook [10] proposed the Johnson–Cook model to establish a rheological stress model under high-temperature, high strain rate, and large strain conditions. This model only considers the individual effects of temperature, strain rate, and strain on rheological stress, with low prediction accuracy, and it is not suitable for deformation under low strain rate and small strain. Liu et al. [11] improved the Johnson–Cook model by considering the coupling effects of strain hardening, strain rate hardening, and thermal softening, and predicted the high-temperature rheological stress behavior of high-strength alloy steel. The results showed that the fit between the simulation and experiment was high, with a maximum relative error controlled within 9.23%. Based on the Arrhenius constitutive model and considering the influence of strain, Hu et al. [12] established a high-temperature constitutive model for 5CrNiMoV alloy and predicted its high-temperature rheological stress behavior at 830 °C~1230 °C. The results showed a correlation coefficient of 0.995. In order to compare the prediction accuracy of the strain-compensated Arrhenius model and the modified Johnson–Cook model, Bu et al. [13] established Arrhenius and Johnson–Cook high-temperature constitutive models for 5CrNiMoV mold steel materials, respectively. The average absolute relative error (AARE) values of the Johnson–Cook and Arrhenius models were 6.82% and 5.71%, respectively. The results indicated that the Arrhenius high-temperature constitutive model had higher accuracy. Deformation at elevated temperatures is an essential step to control the microstructure and performance of the workpiece, and hot deformation is usually used to increase the properties of the material, providing accurate understanding of the thermal behavior of mold materials under diverse deformation parameters [14,15]. The constitutive equation proficiently delineates the deformation characteristics of materials in their plastic state and facilitates stress predictions across varying strain rates and temperatures. To refine the forming process and advance numerical simulation methodologies for the thermal deformation of mold steel, a precise and specialized constitutive model is essential [16]. It is widely acknowledged that Arrhenius-type constitutive models with strain compensation are extensively applied to simulate the hot processing behavior of materials. Therefore, the Arrhenius model is used to predict the flow stress behavior of the new Cr-Mo-V type alloy at high temperatures.

The plasticity of the novel Cr-Mo-V steel can be further evaluated using hot processing maps. Hot processing maps can reflect the processing performance of materials under different deformation conditions, predict the deformation mechanism of materials under different hot processing conditions, and analyze their microstructure characteristics. Prasad et al. proposed the dynamic material model (DMM) [17] to describe the deformation behavior of materials during thermal deformation. Finally, the power dissipation map and instability map were established and overlaid to obtain a hot processing map based on the DMM. At present, this model is widely used to construct hot processing maps for various materials, including aluminum alloys, titanium, magnesium, aluminum, nickel-based alloys, steel, etc., to optimize processing and control their microstructure. Hot-work diagrams can display the microstructure mechanism of metal materials, avoid unstable areas, ensure material stability, and select reasonable processing parameters. Xie et al. [18] established a hot processing map of 25Cr2Ni4MoVA steel based on Prasad’s theory and obtained the optimal working parameters for the alloy, as follows: deformation temperature 1050~1150 °C and strain rate 0.01~0.1 s^−1^. Chen et al. [19] investigated the dynamic recrystallization behavior of 4Cr5MoSiV1 hot-work mold steel. The results indicated that at high temperatures ranging from 1050 °C to 1150 °C, a strain rate of 0.1 to 1 s^−1^ could be achieved, resulting in a completely refined dynamic recrystallization structure. Cheng et al. [20] constructed the hot processing map of a Ti_2_AlNb-based alloy and found that the best processing characteristics were achieved at a temperature of 1050 °C and strain rate of 0.001 s^−1^. The main instability characteristics during hot deformation are mixing, adiabatic shear, and cracks. Therefore, this paper establishes a novel Cr-Mo-V die steel hot processing map based on the DMM to further determine the optimal process parameters.

Hot compression is a dynamic test, which can well characterize the mechanical behavior of materials under different deformation conditions and can also quantitatively characterize the grain size, orientation distribution, and microstructure of the hot-compressed specimens to assess microstructure evolution and lay the groundwork for the establishment of hot processing maps. Therefore, in order to study the high-temperature rheological stress behavior and plasticity of the novel die steel, improve the accuracy of the forging simulation and prediction of the material, determine reasonable parameters of the hot working process of die steel, and improve the microstructure uniformity of the material, in this paper, we conduct hot compression tests on a novel Cr-Mo-V die steel. On the basis of experimental data, the rheological stress behavior of the test steel is analyzed, and the Arrhenius high-temperature constitutive model of novel Cr-Mo-V die steel is established. The accuracy of the constitutive model is further evaluated by comparing the calculated rheological stress with the experimental rheological stress using standard statistical parameters. A 3D hot processing map based on the DMM is established, and the rationality of the process parameters is verified by simulation and trial production.

## 2. Experimental Materials and Methods

**Figure 1:**
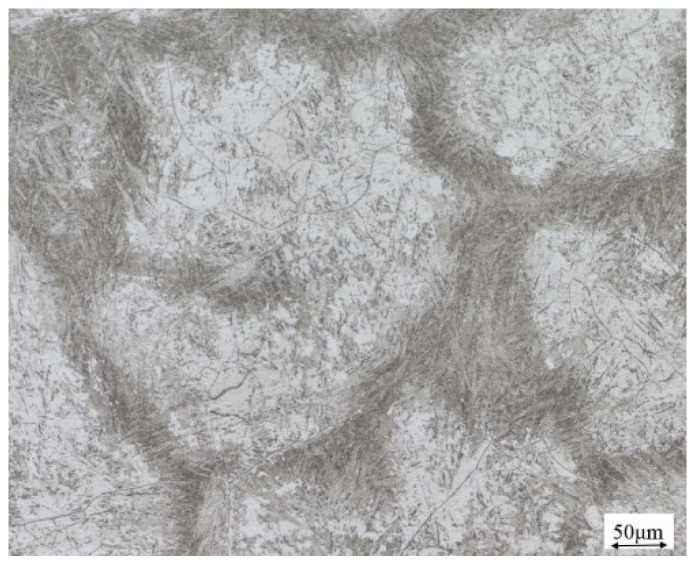
The initial microstructure of novel Cr-Mo-V steel.

**Figure 2:**
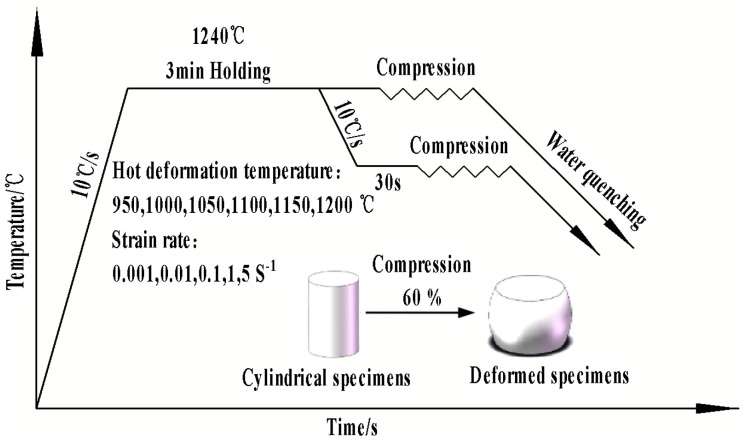
Test process scheme of the test steel.

The material used in this test was 50 kg of small steel ingot smelted in the laboratory, and the chemical composition of the materials used in this experiment is shown in Table 1. The initial state of the microstructure of the experimental material is shown in Figure 1. The sample size of Φ8 × 12 mm was cut using a molybdenum wire cutting machine, and the high-temperature compression test was completed on the Gleeble-1500D thermal simulation testing machine (Dynamic Systems Inc., Poestenkill, NY, USA), with selected deformation temperatures of 950, 1000, 1050, 1100, 1150, and 1200 °C, strain rates of 0.001, 0.01, 0.1, 1, and 5 s^−1^, and a deformation amount of 60% (maximum true strain was 0.916). The detailed process is shown in Figure 2. We tested one sample for each parameter to obtain valid data. After the experiment was completed, the cylindrical specimen was cut along the axis, and half of it was polished and corroded to obtain a metallographic specimen. The microstructure under different deformation conditions was observed using an M_2_M optical microscope.

By employing finite element simulation technology, a finite element simulation for the forging of the novel Cr-Mo-V hot-work die steel was established via Forge^®^ simulation software (https://www.transvalor.com/en/forge (accessed on 27 November 2024)) to predict the parameters, such as the temperature and stress fields during the forging process of the hot-work die steel.

## 3. High-Temperature Rheological Stress Model of a Novel Cr-Mo-V Hot-Work Die Steel

### 3.1. True Stress–Strain Curve

The rheological stress value of the metal materials during deformation was determined by both work hardening and dynamic softening. Work hardening led to the accumulation of dislocations inside the material, an increase in dislocation density, and a rapid increase in rheological stress. Dynamic softening led to a decrease in dislocation density, a decrease in distortion energy, and a decrease in rheological stress. In the early stage of deformation, the work hardening effect was significant, and the dislocation density increased with increasing strain, resulting in a rapid increase in rheological stress. When the true stress reached the critical value, the material underwent dynamic recovery, gradually weakening the influence of work hardening, and ultimately, the true stress–strain curve tended to flatten, which is typical of a dynamic recovery curve. For dynamic recrystallization curves, when the critical strain was reached, recrystallization began to occur inside the material. As the strain increased, the dynamic softening caused by recrystallization greatly weakened the work hardening effect, causing the flow stress to peak and then begin to decrease. The deformation continued to increase, the dynamic softening and work hardening reached dynamic equilibrium, and the flow stress gradually tended to stabilize.

**Figure 3:**
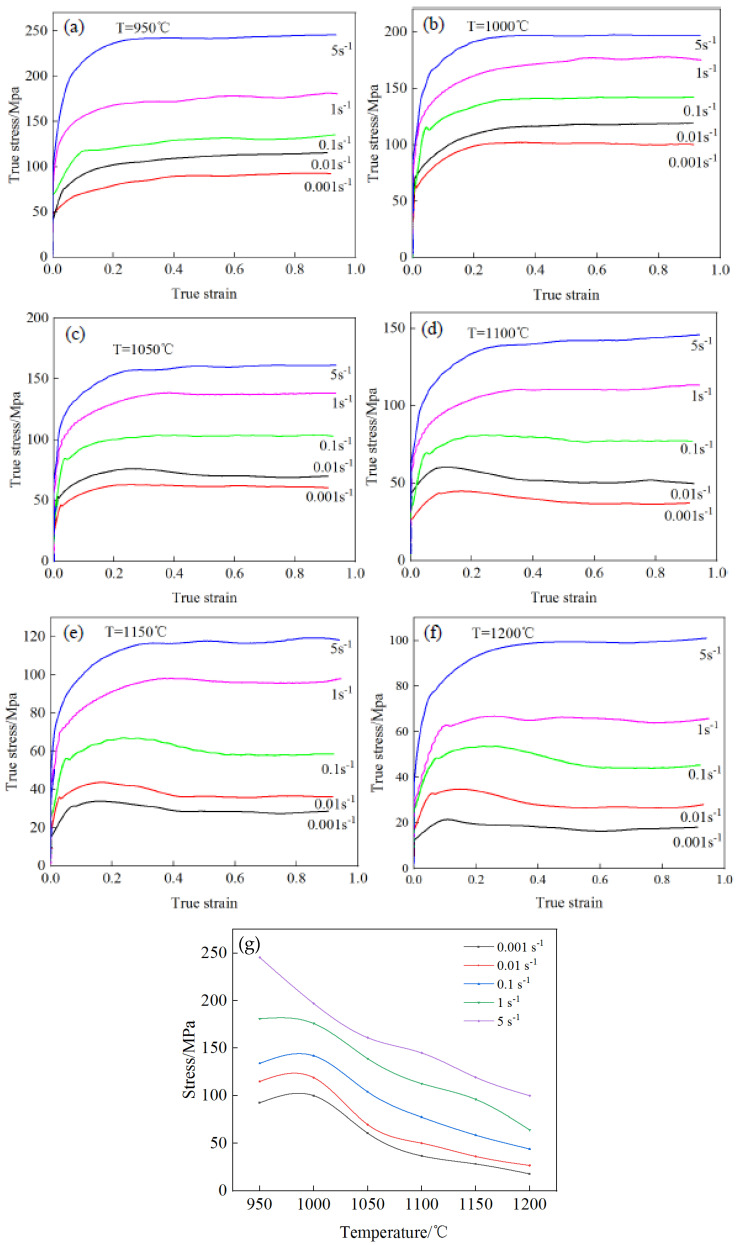
True stress–strain and temperature–stress curves for the test steel under different temperatures and strain rates. (**a**) 950 °C; (**b**) 1000 °C; (**c**) 1050 °C; (**d**) 1100 °C; (**e**) 1150 °C; (**f**) 1200 °C; (**g**) Curve of temperature-stress.

Figure 3 shows the true stress–strain curve and temperature–stress curve of the novel Cr-Mo-V steel compressed under different deformation conditions. From the graph, it can be seen that under the deformation conditions of this experiment, the flow stress curve of the novel Cr-Mo-V steel showed two types: dynamic recovery type and dynamic recrystallization type. The deformation temperature was below 1050 °C, and the strain rate was below 0.1 s^−1^. The rheological stress showed a trend of rapid increase, slow growth, and stabilization with the increase in true strain, exhibiting typical dynamic recovery characteristics. When the deformation temperature exceeded 1050 °C and the strain rate was below 0.1 s^−1^, the stress increased rapidly with the increase in strain. After that, the stress showed a small decrease and gradually stabilized, and the dynamic recrystallization characteristics were more significant. As shown in Figure 3g, at the same deformation temperature, the higher the strain rate, the higher the peak stress and strain at which the peak stress was reached. This is because as the strain rate increased, the degree of work hardening increased, exhibiting an increase in peak stress and strain. At the same strain rate, the higher the deformation temperature, the smaller the peak stress. This is mainly because as the deformation temperature increased, the dynamic softening effect was enhanced, showing a decrease in peak stress and steady-state stress.

**Figure 4:**
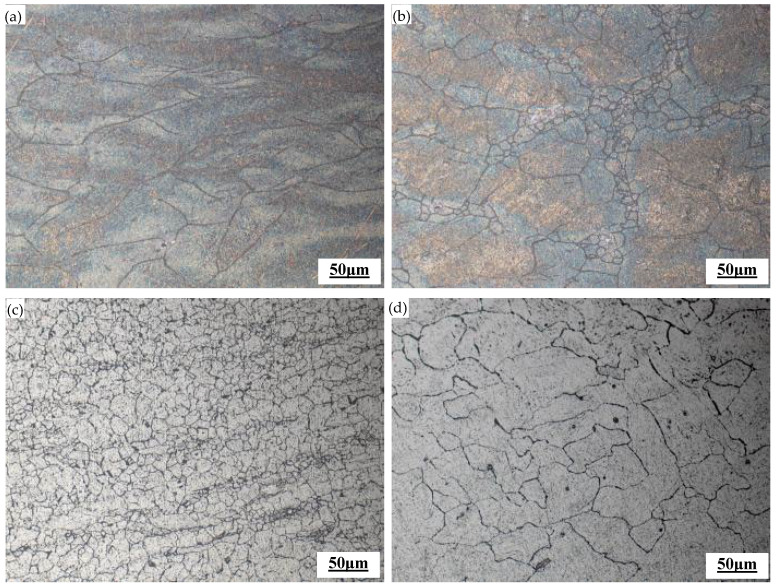

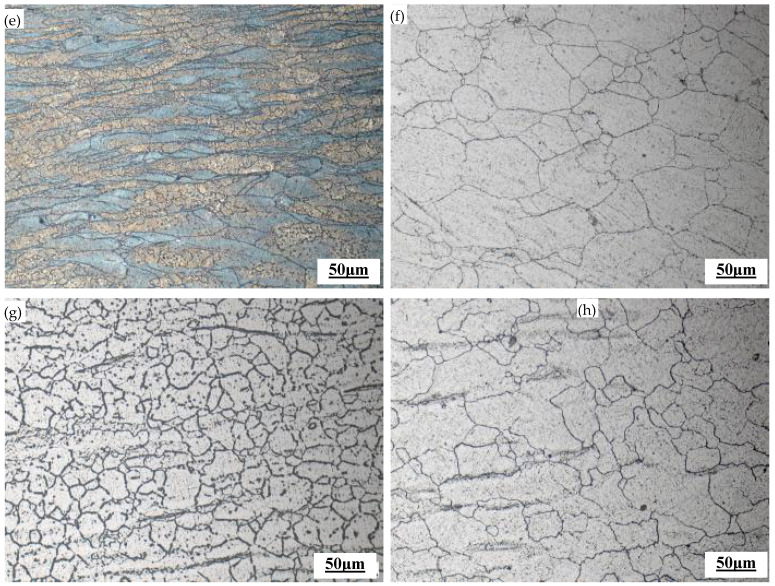
Microstructure under different deformation conditions: (**a**) 950 °C and 0.1 s^−1^, (**b**) 1050 °C and 0.1 s^−1^, (**c**) 1150 °C and 0.1 s^−1^, (**d**) 1200 °C and 0.1 s^−1^, (**e**) 1100 °C and 5 s^−1^, (**f**) 1100 °C and 0.1 s^−1^, (**g**) 1100 °C and 0.01 s^−1^, and (**h**) 1100 °C and 0.001 s^−1^.

Based on the stress–strain curve, two sets of compressed specimens under different deformation conditions were selected for microstructure analysis, as shown in Figure 4. At the same strain rate, as the deformation temperature increased, the number of recrystallized grains gradually increased, and the degree of recrystallization increased. When the strain rate was 0.1 s^−1^ and the deformation temperature was 950 °C, due to the lower temperature, the original austenite grains showed a slightly elongated trend, as shown in Figure 4a. When the deformation temperature reached 1050 °C, the microstructure was composed of fine recrystallized grains and original austenite grains. Fine recrystallized grains formed along the boundaries of the original austenite, as shown in Figure 4b, and the recrystallized grain volume present was 40.9%. When the deformation temperature continued to rise to 1150 °C, all the original austenite grains are replaced by fine recrystallized grains, and complete dynamic recrystallization occurred (consistent with the stress–strain curve in Figure 3). With an average grain size of about 19.2 μm, the grain size class can reach 7.5. As shown in Figure 4c, the recrystallized grain volume present was 98.9%. When the deformation temperature was 1200 °C, the new grains that underwent dynamic recrystallization were significantly coarsened, with an average grain size of 63.1 μm, as shown in Figure 4d. When the deformation temperature was 1100 °C and the strain rate was 5 s^−1^, the grains were significantly elongated and the material underwent dynamic recovery, as shown in Figure 4e. As the strain rate changed from 0.1 s^−1^ to 0.01 s^−1^ to 0.001 s^−1^, the grains exhibited a pattern of incomplete dynamic recrystallization, the grain size class changed in order from 3.0 to 6.0 to 3.5, and the degree of recrystallization was inversely proportional to the strain rate. With the change in the strain rate from 0.1 s^–1^ to 0.01 s^–1^, the recrystallization volume present increased from 39.1% to 99.5%, as shown in Figure 4f,g. When the strain rate decreased from 0.01 s^−1^ to 0.001 s^−1^, the average grain size increased from 23.9 μm, when dynamic recrystallization had just completed, to 73.6 μm, and the grain size class decreased from 7.0 to 3.5, as shown in Figure 4g,h.

The study of the microstructure under different deformation conditions showed that at a certain strain rate, the higher the deformation temperature, the greater the internal thermal activation energy of the material, the intensified atomic diffusion, the rapid migration of grain boundaries, and the promotion of nucleation and growth of recrystallized grains. The higher the deformation temperature, the smaller the peak stress, and the lower the corresponding critical strain [21], which was conducive to the dynamic recrystallization process. When the material reached the deformation temperature of complete dynamic recrystallization, the strain rate was small, and there was still enough time left after the material underwent dynamic recrystallization to promote the growth of recrystallized grains. On the contrary, a relatively large strain rate is more conducive to grain refinement.

The rheological stress during hot deformation can characterize the plasticity of the materials, and the magnitude of stress is closely related to the strain rate, deformation temperature, deformation amount, and alloy composition [22,23,24]. Therefore, in order to fully consider the influence of the strain rate and deformation temperature on the high-temperature rheological stress of the novel Cr-Mo-V steel, it is necessary to establish a material rheological stress constitutive model. In the 1960s, the Arrhenius equation proposed by Lian et al. was able to better describe the three factors ($\overset{\cdot }{\varepsilon }$, T, and σ) and the relationship between them [25,26]:

(1) $$\dot{\varepsilon }=AG(\sigma )\times \exp(\frac{-Q}{RT})$$

In the formula, $\overset{\cdot }{\varepsilon }$ is the strain rate, s^−1^; T is the deformation temperature, K; σ is the peak stress, MPa; Q is the activation energy of thermal deformation, J/mol; R is the ideal gas constant, usually taken as 8.314 J/(K·mol); α, β, N, and A are material constants, where α = β/n.

In Equation (1):

(2) $$G(\sigma )=\left\{\begin{array}{c} \sigma^{n1}\;(\alpha \sigma <0.8)\\ exp(\beta \sigma )\;(\alpha \sigma >1.2)\\ {\left(sinh(\alpha \sigma )\right)}^{n}\;All \end{array}\right.$$

Substituting Equation (2) into Equation (1), we obtained Equation (3):

(3) $$\dot{\varepsilon }=\left\{\begin{array}{c} {A\sigma }^{n1}\times exp(-\frac{Q}{RT})\;(\alpha \sigma <0.8)\\ Aexp(\beta \sigma )\times exp(-\frac{Q}{RT})\;(\alpha \sigma >1.2)\\ A{\left(sinh(\alpha \sigma )\right)}^{n}\times exp(-\frac{Q}{RT})\;All \end{array}\right.$$

When ασ is large, $sinh(\alpha \sigma )=\frac{e^{\alpha \sigma }-e^{-\alpha \sigma }}{2}$, where e^−ασ^ approaches 0, then $\dot{\varepsilon }=A\frac{e^{\alpha \sigma n}}{2^{n}}exp(-\frac{Q}{RT})=A^{\prime }exp(\alpha n\sigma )exp(-\frac{Q}{RT})$, in the formula. A′ = A/2^n^, and α = β/n_1_. We took the natural logarithm at both ends of Equation (3) to obtain Equations (4)–(6), respectively:

(4) $$\ln\dot{\varepsilon }=lnA_{1}+n_{1}ln\sigma -\frac{Q}{RT}\;\alpha \sigma <0.8$$

(5) $$\ln\dot{\varepsilon }=lnA_{2}+\beta \sigma -\frac{Q}{RT}\;\alpha \sigma >1.2$$

(6) $$\ln\dot{\varepsilon }=lnA+nln(sinh(\alpha \sigma ))-\frac{Q}{RT}\;All$$

**Figure 5:**
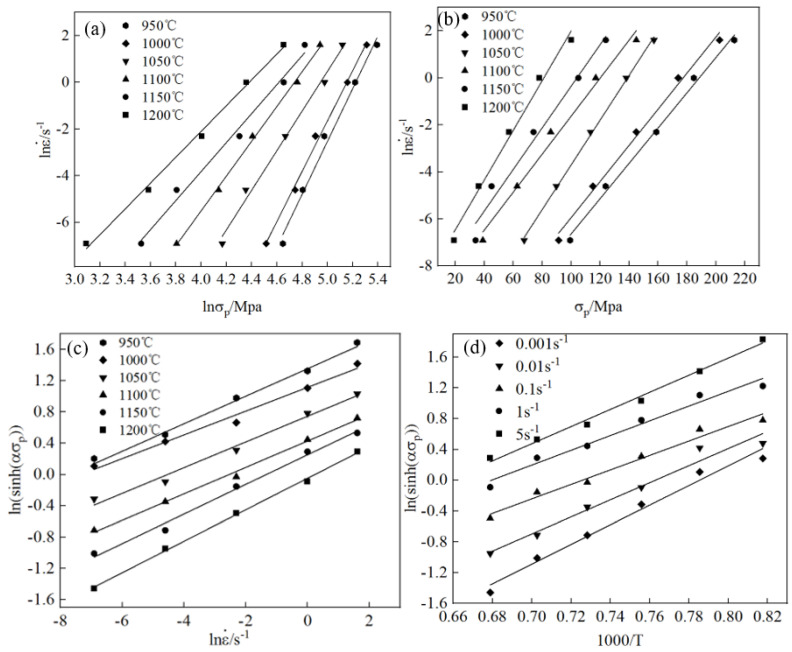
Fitting curves for test steel under different temperatures and strain rates: (**a**) ln$\overset{\cdot }{\varepsilon }$–lnσp, (**b**) ln$\overset{\cdot }{\varepsilon }$–σp; (**c**) ln(sinh(ασ))–ln$\overset{\cdot }{\varepsilon }$, and (**d**) ln(sinh(ασ))–1000/T.

According to Equation (4), at the same temperature, a ln$\overset{\cdot }{\varepsilon }$ and lnσ linear relationship was formed, and its relationship curve is shown in Figure 5a, describing the relationship between ln$\overset{\cdot }{\varepsilon }$ and lnσ at different temperatures. The relationship is determined by linear fitting to obtain the slope of the straight line at six temperatures and taking the average, n_1_ = 8.288665.

Similarly, for ln$\overset{\cdot }{\varepsilon }$–σ, the fitting relationship curve is shown in Figure 5b, where for the slope of the line, l/β = 11.498086, β = 0.086971, and α = 0.010493.

For the strain rate in Equation (6), taking partial derivatives of ε and 1/T, respectively, yielded: $\frac{1}{n}=\frac{\partial ln[sinh(\alpha \sigma )]}{\partial ln\dot{\varepsilon }},\frac{Q}{nR}=\frac{\partial ln[sinh(\alpha \sigma )]}{\partial (1/T)}=K$. The relationship curves of ln(sinh(asp))–ln $\overset{\cdot }{\varepsilon }$ and ln(sinh(asp))–ln1000/T are shown in Figure 5c and Figure 5d, respectively. Through linear fitting, n = 5.704018 and K = 11.397144 were obtained. Therefore, for the hot deformation activation energy of the tested steel, Q = nRK = 540,489.104 J/mol.

When a material undergoes high-temperature plastic deformation, the parameter Z in Whiteman and Sellers’ theory can accurately reflect the relationship between the true stress magnitude, strain rate, and deformation temperature:

(7) $$Z=\dot{\varepsilon }exp(\frac{Q}{RT})$$

Among them, Z is the Zener–Holomon parameter, which is also the strain rate for temperature compensation. Considering the deformation activation energy Q, Z can be calculated for different strain rates and deformation temperatures.

Substituting Equation (7) into Equation (3) yielded the relationship between Z and σ:

(8) $$Z=A[sinh(\alpha \sigma ){]}^{n}$$

Taking the derivative on both sides of Equation (8) yielded:

(9) lnZ=nln(sinh(ασ))+lnA

**Figure 6:**
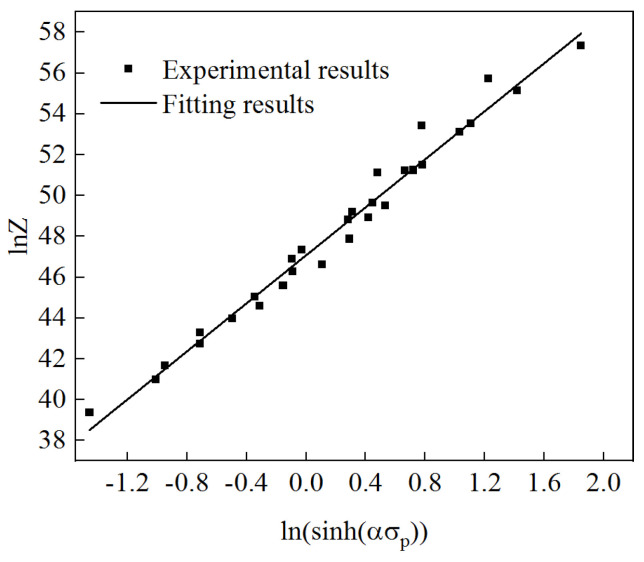
Fitting curve of lnZ–ln(sinh(ασ)).

From this, it can be seen that for lnZ and ln (sinh)(ασ_p_), a linear relationship was formed, and the relationship curve is shown in Figure 6. Via fitting, the slope n = 5.87594 was obtained, which is similar to the slope n = 5.704018 obtained from Figure 5c. If the intercept lnA is 47.07874, then A = 2.78278 × 10^20^.

Adding A, α, n, and Q into Equation (3) separately, $\dot{\varepsilon }=A[sinh(\alpha \sigma ){]}^{n}exp(-\frac{Q}{RT})$ can be used to obtain the thermal deformation constitutive equation of the tested steel:

(10) $$\dot{\varepsilon }=2.78278\times 10^{20}{\left[\sinh(0.01049\sigma )\right]}^{5.87594}\times \exp(\frac{-540489.104}{RT})$$

### 3.2. Solution and Verification of Parameters of the High-Temperature Rheological Model

Material model parameters are closely related to true strain, and the thermal activation energy and material constant change with strain changes. In order to improve the limitation of the traditional Arrhenius equation in the flow stress prediction process, the Arrhenius model based on strain compensation was used in this paper to describe the flow stress of the material [27,28,29]. According to the hot compression data, a point (true stress–true strain) was selected every 0.05 interval in the strain range of 0.05–0.80, and the values of α, n, Q, and lnA without true strain were calculated by using the fitting model material parameters, as shown in Table 2. The relationship between the material constant and true strain was drawn from the data in the table, as shown in Figure 6. Equation (11) was obtained by using eight-polynomial fitting. The corresponding polynomial coefficients are shown in Table 3.

**Figure 7:**
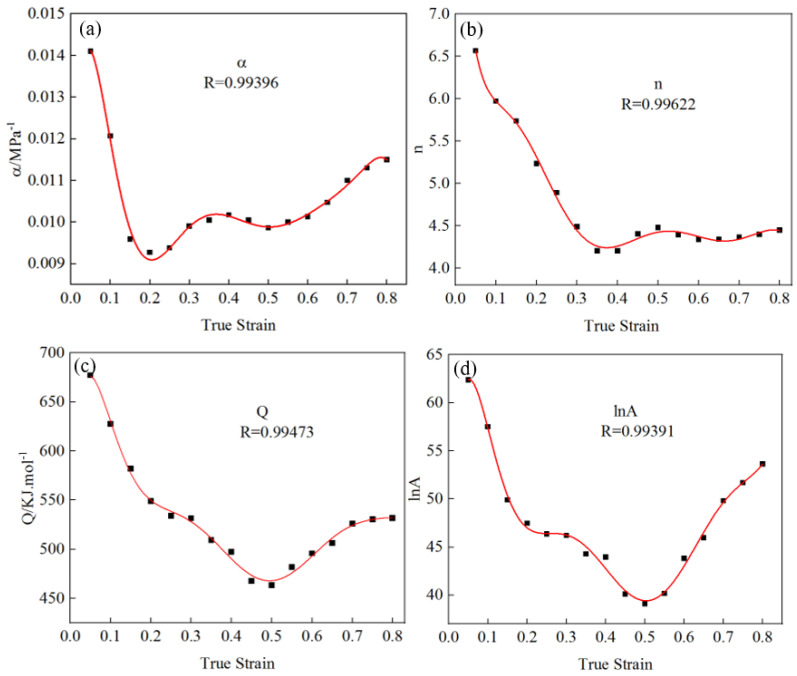
Relationship between material parameters and true strain and the polynomial fitting curve of eight degrees: (**a**) α, (**b**) n, (**c**) Q, and (**d**) lnA.

It can be seen from Figure 7 that the fitting correlation coefficients of α, n, Q, and lnA values with true strain were 0.99396, 0.99622, 0.99473, and 0.99391, respectively. The fitting results were better, indicating that the curve can truly reflect the relationship between the material parameters α, n, Q, and lnA and true strain.

(11) $$\begin{array}{c} \alpha =\alpha_{0}+\alpha_{1}\varepsilon +\alpha_{2}\varepsilon^{2}+\alpha_{3}\varepsilon^{3}+\alpha_{4}\varepsilon^{4}+\alpha_{5}\varepsilon^{5}+\alpha_{6}\varepsilon^{6}+\alpha_{7}\varepsilon^{7}+\alpha_{8}\varepsilon^{8}\\ \begin{array}{c} n=n_{0}+n_{1}\varepsilon +n_{2}\varepsilon^{2}+n_{3}\varepsilon^{3}+n_{4}\varepsilon^{4}+n_{5}\varepsilon^{5}+n_{6}\varepsilon^{6}+n_{7}\varepsilon^{7}+n_{8}\varepsilon^{8}\\ Q=Q_{0}+Q_{1}\varepsilon +Q_{2}\varepsilon^{2}+Q_{3}\varepsilon^{3}+Q_{4}\varepsilon^{4}+Q_{5}\varepsilon^{5}+Q_{6}\varepsilon^{6}+Q_{7}\varepsilon^{7}+Q_{8}\varepsilon^{8}\\ lnA=A_{0}+A_{1}\varepsilon +A_{2}\varepsilon^{2}+A_{3}\varepsilon^{3}+A_{4}\varepsilon^{4}+A_{5}\varepsilon^{5}+A_{6}\varepsilon^{6}+A_{7}\varepsilon^{7}+A_{8}\varepsilon^{8} \end{array} \end{array}$$

Equation (11) was substituted into Equation (9), and according to hyperbolic sine transformation, the high-temperature flow stress model of the test steel under different strain rates (0.0001–5 s^−1^) and different deformation temperatures (950–1200 °C) can be obtained. The constitutive equation is as follows:

(12) $$\left\{\begin{array}{c} \sigma =\frac{1}{\alpha (\varepsilon )}ln\left\{({\frac{Z(\varepsilon )}{A(\varepsilon )})}^{1/n(\varepsilon )}+[({\frac{Z(\varepsilon )}{A(\varepsilon )})}^{2/n(\varepsilon )}+1{]}^{1/2}\right\}\\ Z(\varepsilon )=\dot{\varepsilon }exp(\frac{Q(\varepsilon )}{RT}) \end{array}\right.$$

**Figure 8:**
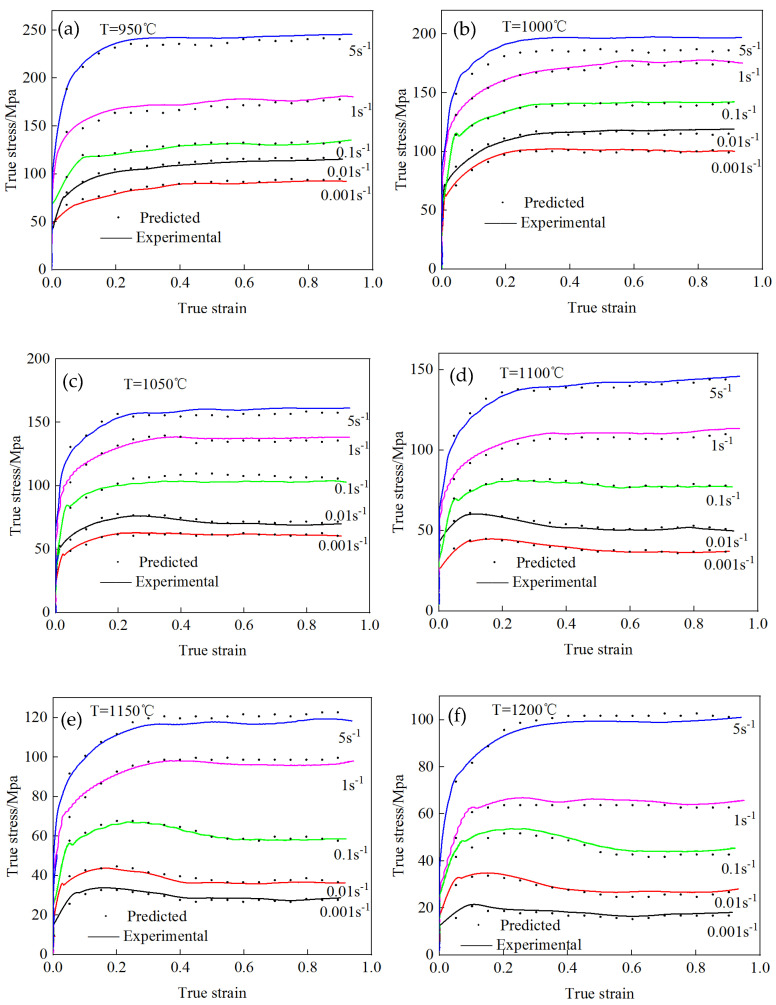
Theoretical and experimental values under different deformation conditions. (**a**) 950 °C; (**b**) 1000 °C; (**c**) 1050 °C; (**d**) 1100 °C; (**e**) 1150 °C; (**f**) 1200 °C.

Equation (12) was used to calculate the true stress–true strain values under different deformation conditions. The comparison results between the test stress values and the calculated stress values under different strains are shown in Figure 8. Under the deformation conditions of a deformation temperature of 950–1200 °C and strain rate of 0.001–0.1 s^−1^, the test values were basically the same as the calculated values, and the deformation temperature was lower than 1050 °C. When the strain rate was 5 s^−1^, the calculated value was slightly lower than the test value. When the deformation temperature exceeded 1100 °C, the strain rate was greater than 1 s^−1^, the calculated value was slightly higher than the test value, and the overall error was small.

To further evaluate the accuracy of the strain-based compensation model, the correlation coefficient (R) and absolute relative error (AARE) were used to quantitatively evaluate the model’s accuracy. The expressions of parameters R and AARE are shown in Equation (13):

(13) $$\left\{\begin{array}{c} R=\frac{\sum_{i=1}^{n}\left(P_{i}-\bar{P})(E_{i}-\bar{E}\right)}{\sqrt{\sum_{i=1}^{n}(P_{i}-\bar{P}{)}^{2}\sum_{i=1}^{n}(E_{i}-\bar{E}{)}^{2}}}\\ AARE=\frac{100\%}{n}\sum_{i=1}^{n}\left|\frac{P_{i}-E_{i}}{E_{i}}\right| \end{array}\right.$$

**Figure 9:**
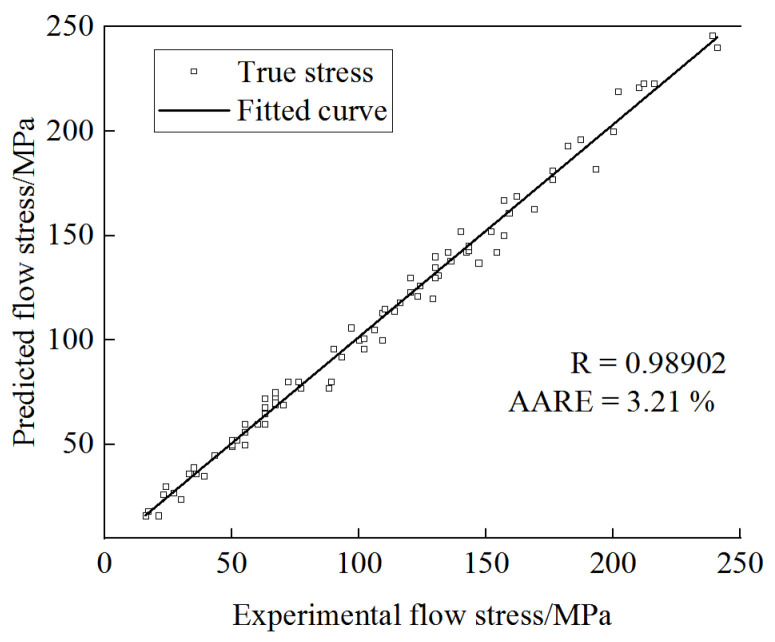
The correlation curve of theoretical and experimental values.

In these equations, P_i_ and E_i_ represent calculated values and test values, respectively, $\bar{P}\;and\;\bar{E}$ represent the average values of P_i_ and E_i_, respectively, and n represents the total number of data points. The correlation between experimental and calculated flow stress values under all deformation conditions is shown in Figure 9. The R and AARE were 0.98902 and 3.21%, indicating that the strain-compensation-based Arrhenius model can better predict flow stress during high-temperature deformation. It can provide a theoretical basis for developing a reasonable production and forming process for large die steel forgings.

### 3.3. 3D Hot Processing Map of the Novel Cr-Mo-V Die Steel

The hot processing map is the most effective method to guide the thermal deformation of materials and control the microstructure. In this paper, we systematically studied the forming conditions of the novel Cr-Mo-V die steel. According to the thermodynamic theory of irreversible process, the hot processing map of the test steel was established by using the dynamic material model (DMM) method, and the microstructure evolution under different thermal deformation conditions was combined. The optimum plastic forming process was designed. The power dissipation map was obtained by calculating the power dissipation coefficient, and the instability map was constructed based on the instability criterion. The hot processing map of the test steel based on the DMM can be obtained by superposing the power dissipation map and the instability map. The hot processing map can not only clearly show the distribution of the power dissipation coefficient under different deformation conditions, but can also effectively judge the safe zone and instability zone in the forming process of the material, avoid the flow instability defects caused by cracks and adiabatic shear bands in the forming process of the die steel, optimize the deformation process parameters, and improve the yield. It provides theoretical guidance for the formulation of hot deformation processes of this kind of die steel in actual production.

The main purpose of constructing the hot processing map based on the DMM was to optimize the hot processing parameters and control the microstructure. According to the dynamic material model theory, the power dissipation, P, of the workpiece during thermal deformation is divided into two parts [30,31]: one is the dissipation G, generated by the plastic deformation of the workpiece, and the other is the dissipation J, generated by the microstructure evolution inside the workpiece, that is:

(14) $$p=\sigma \overset{•}{\varepsilon }=G+J=\int_{0}^{\overset{•}{\varepsilon }}\sigma d\overset{•}{\varepsilon }+\int_{0}^{\sigma }\overset{•}{\varepsilon }d\sigma$$

Under a certain deformation temperature, the relationship between the strain rate and flow stress can be expressed as follows:

(15) $$\sigma =K{\overset{•}{\varepsilon }}^{m}$$

In Equation (15), K is the material constant, and m is a strain-rate-sensitive factor, which can be used to describe the proportion of dissipation generated by the two methods:

(16) $$m=\frac{dJ}{dG}=\frac{\overset{•}{\varepsilon }d\sigma }{\sigma d\overset{•}{\varepsilon }}={\left[\frac{\partial (\ln\sigma )}{\partial (\ln\overset{•}{\varepsilon })}\right]}_{\overset{•}{\varepsilon }T}$$

The power dissipation coefficient represents the change in energy during the evolution of the microstructure and is defined as:

(17) $$\eta =\frac{J}{J_{\max}}$$

When m is 1, the J value reaches the maximum, and the material is in an ideal linear dissipation state [32,33], that is:

(18) $$J_{\max}=\frac{1}{2}\sigma \overset{•}{\varepsilon }$$

Based on Prasad’s theory, the m value at a fixed temperature is independent of the strain rate, and the power dissipation coefficient, η, of the material is obtained by combining Equations (14)–(18):

(19) $$\eta =\frac{2m}{m+1}$$

In view of the thermal deformation process of steel materials, Prasad [17] believed that the thermal deformation instability condition can be expressed as:

(20) $$\xi (\overset{•}{\varepsilon })=\frac{\partial \log\frac{m}{m+1}}{\partial \log\overset{•}{\varepsilon }}+m<0$$

**Figure 10:**
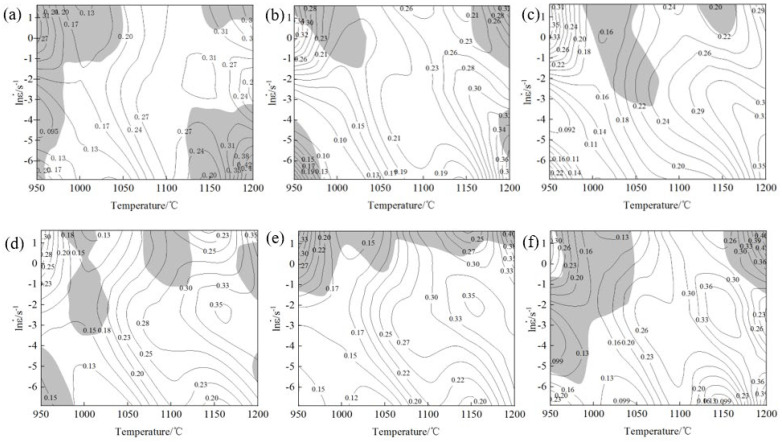
Hot processing maps at different strains: (**a**) 0.05, (**b**) 0.2, (**c**) 0.35, (**d**) 0.5, (**e**) 0.65, and (**f**) 0.8.

Figure 10 shows the hot processing map of the novel Cr-Mo-V die steel under different strains, in which the gray area indicates that the test steel was prone to flow instability during the thermal deformation process, and the contour value indicates the distribution value of the power dissipation coefficient. It can be seen that most of the instability regions of the material occurred in the regions of low temperature and high strain rate and high temperature and high strain rate, and most of the peak power dissipation regions occurred in the regions of high temperature and low strain rate and middle strain rate. There was overlap between the instability regions and the peak power dissipation regions.

Because the 2D hot processing map only considers the influence of the strain rate and temperature on the plasticity of the material, the strain was not included in the analysis. However, strain, as an important component of deformation conditions, is also sensitive to the plasticity of materials. Therefore, in order to better analyze the influence of strain, temperature, and strain rate on the plasticity of the new Cr-Mo-V die steel, a three-dimensional hot processing map considering strain was established in this paper.

**Figure 11:**
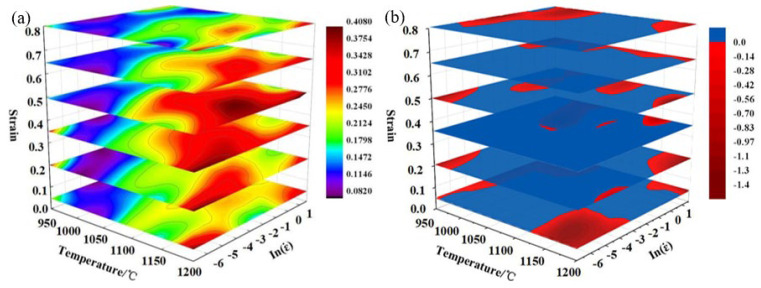
The 3D power dissipation map (**a**) and 3D instability map (**b**).

Figure 11a shows the 3D power dissipation map of the test steel under different strains. It can be seen from the map that the deformation region of the peak power dissipation first increased and then decreased with the increase in strain, and the region changed from high temperature and a low strain rate to medium–high temperature and a medium strain rate. When the strain reached 0.3–0.5, the peak deformation region of power dissipation was the largest. The main reasons are as follows: In the early stage of deformation, the strain was small, the work hardening effect was large, the internal atomic activity was intensified at high temperature, the critical dislocation density of the material was small, and the dynamic recrystallization could be fully dynamic at a low strain rate. The cumulative dislocation density increased with the increase in strain. When the dislocation density exceeded the critical dislocation density, dynamic recrystallization occurred. At a high temperature and low strain rate, coarsening of dynamic recrystallized grains occurred due to a higher deformation temperature and longer deformation time. At a medium–high temperature and medium strain rate, the deformation time was relatively short, and the recrystallization grains were not coarsened.

Combined with the 3D instability map in Figure 11b, the red region is the instability region. It can be seen from the figure that when the strain was 0.05, the dissipative instability coefficient at a high temperature and low strain rate was higher, but it belonged to the instability region in the instability map, mainly because at the initial stage of strain, the work hardening effect was large, the proportion of recovery and dynamic recrystallization inside the material was small, and the storage energy of thermal deformation could not be consumed in time, which led to instability, and the thermal deformation process should avoid this area.

The instability and power dissipation were not only related to the strain rate and deformation temperature, but also closely related to the strain. With the increase in the strain, the instability region decreased first and then increased. When the strain was low, the deformation instability region of the steel was mainly concentrated in the low-temperature and high strain rate region and the high-temperature and high strain rate region. The instability zone was mainly in the regions with a temperature of 950~1048 °C and strain rate of 0.32~5 s^−1^ and temperature of 1122~1200 °C and strain rate of 0.001~0.039 s^−1^. As the strain increased, the room-temperature region began to move to the region with a low and medium temperature and low strain rate, as shown in Figure 9. The temperature of the unstable region was mainly concentrated in the region of temperature 992~1075 °C and strain rate 0.042~5 s^−1^. When the strain reached 0.5, four instability regions appeared, but the area of the instability region was small. When the strain continued to increase to 0.65, the temperature loss region was concentrated in the high strain rate region (950~997 °C, 0.21~5 s^−1^ and 997~1200 °C, 1.25~5 s^−1^). When the strain increased to 0.8, the area of the temperature region increased and diffused to the medium–low-temperature region and the high-temperature region with low strain rate (1148~1200 °C, 0.37~5 s^−1^ and 950~1046 °C, 0.0028~5 s^−1^). In principle, the area outside the deformation instability zone in the figure above is a deformation safety zone, but it is generally believed that the region with power dissipation ≥ 0.3 has a better thermal processing performance. It can be seen that when the strain was 0.05~0.35, the temperature was 1100~1175 °C, and the strain rate was 0.001~0.009 s^−1^—this was the first suitable processing zone. There was a peak power dissipation value of 0.42. When the strain was 0.45~0.65, the temperature was 1100~1200 °C, and the strain rate was 0.024~0.33 s^−1^. This second suitable processing zone had a peak power dissipation value of 0.36. Usually, the high η region in the safe zone is the first selection of the steel for hot processing, but the optimal hot processing region must also be determined in combination with the microstructure evolution.

**Figure 12:**
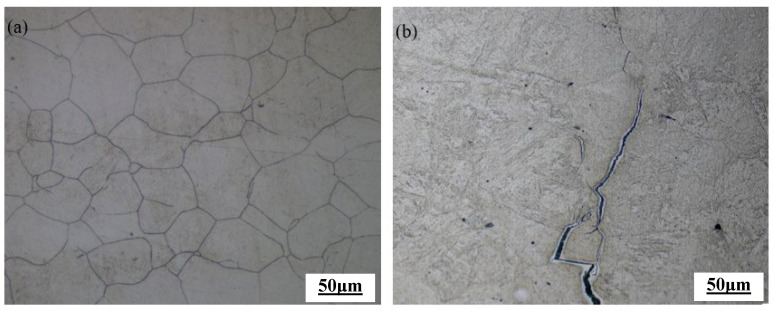
Microstructures of the safe zone and unstable zone of the novel Cr-Mo-V steel: (**a**) 1100 °C-0.1 s^−1^ and (**b**) 1000 °C-1 s^−1^.

Figure 12 shows the metallographic structures of the novel Cr-Mo-V steel safe zone and unstable zone. As illustrated in Figure 12a, under deformation conditions of 1100 °C and a strain rate of 0.1 s^−1^, the microstructures of the hot-compressed specimen in the axial large deformation zone exhibited significant dynamic recrystallization in the novel Cr-Mo-V alloy, and the original austenite grains were all replaced by fine and equiaxial new grains. In contrast, as depicted in Figure 12b, under deformation conditions of 1000 °C and a strain rate of 1 s^−1^, the microstructures observed in the axial large deformation zone revealed noticeable cracking phenomena along with unstable deformation structures, and crack initiation and expansion along the austenite grain boundary until cracking. An unstable deformation structure appeared. The safe zone structure and unstable structure of the alloy indicated that the hot processing map could well describe the hot workability of the novel Cr-Mo-V steel. The first suitable processing zone was a strain of 0.05~0.3, temperature of 1100~1175 °C, and strain rate of 0.001~0.009 s^−1^. The second suitable processing zone was a strain of 0.45~0.65, temperature of 1100~1200 °C, and strain rate of 0.0024~0.33 s^−1^.

### 3.4. Industrial Verification of Plasticity of the Novel Cr-Mo-V Die Steel Based on Finite Element Simulation

**Figure 13:**
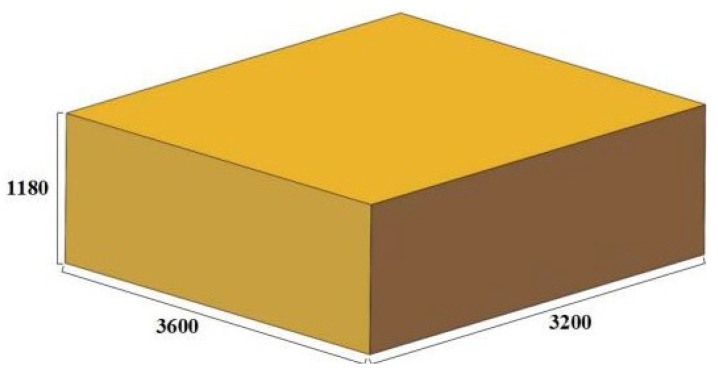
The size diagram of 183 t module forging.

In order to verify the validity of the 3D hot processing map, the Forge finite element simulation software was used to predict the forging process of the 3600 × 3200 × 1180 mm module and the actual production. The finished size is shown in Figure 13.

**Figure 14:**
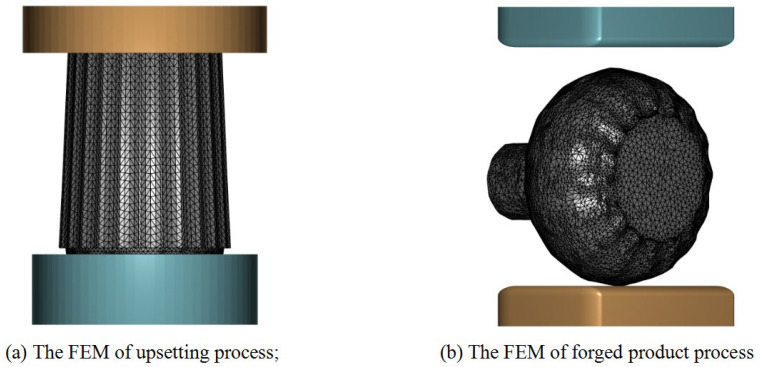
The FEM of billet and dies of the 183 t ingot forging process.

The established high-temperature constitutive model of Cr-Mo-V steel was incorporated into the simulation software. The friction type between the billet and die was set as Coulomb friction with a coefficient of 0.7. The heat exchange coefficient between the billet and air was 10 W(m^2^·K)^−1^, while the heat exchange coefficient between the billet and die was 2000 W(m^2^·K)^−1^. The damage distribution of the die steel during forging was analyzed and predicted using the Cockcroft–Latham normalized criterion damage model. A three-dimensional FEM (finite element model) of an ingot (183 t) between upper and lower dies was constructed and imported into the Forge simulation software. The number of mesh elements in the blank was set to 180,000, as depicted in Figure 14.

**Figure 15:**
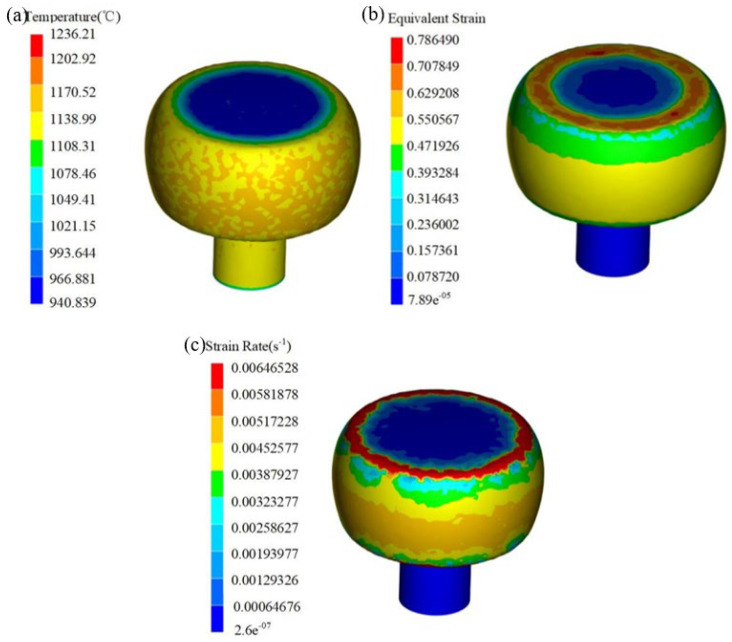
Simulation results of upsetting of the novel Cr-Mo-V die steel of 183 t.

The module selected a 183 t ingot for forging, with an ingot height of 3800 mm and average diameter of 2580 mm. First of all, based on the results of the 3D hot processing map, the upsetting process was determined to be within the second suitable processing area, where the ingot was upset to H = 1800 mm, φ = 3600 mm, the overall strain was about 0.526, considering that it takes 3~5 min to prepare the tool, and the oven temperature was set to 1240 °C. Then, the upsetting process was simulated and predicted. In Figure 15a–c, the temperature field and the strain rate field of the upsetting simulation are shown, respectively. It can be seen that the module was upsetting to the technological requirements. The forging temperature was 1108~1170 °C, the strain was 0.47~0.55, and the strain rate was 0.0032~0.006 s^−1^. It is shown that the forging process parameters were all in the second suitable processing zone. 

**Figure 16:**
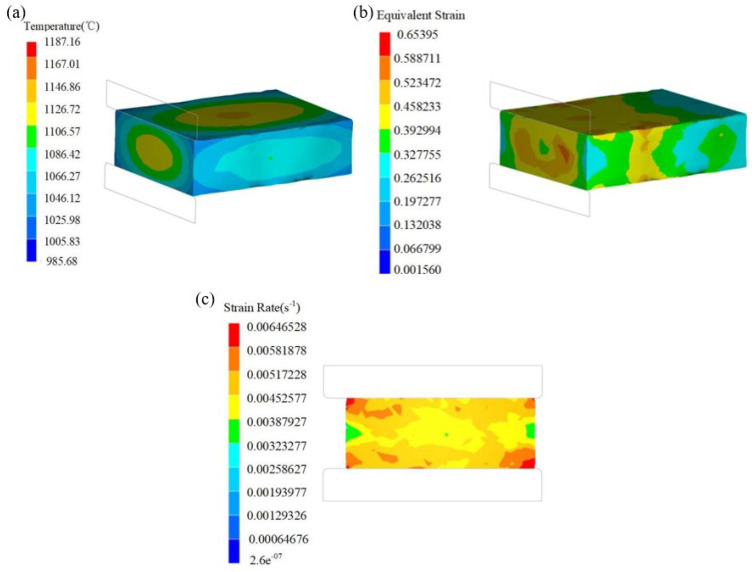
Simulation results of the novel Cr-Mo-V die steel of 183 t.

Next, the first suitable processing zone in the 3D hot processing map was selected, and the finished product was simulated. The novel Cr-Mo-V die steel billet temperature was also set at 1240 °C, and the forging press of each anvil was controlled at 20%; that is, the strain was controlled at 0.2. The simulation results of the finished product produced by the module forging are shown in Figure 13. Figure 16a–c, respectively, show the temperature field, strain field, and strain rate field of the finished product simulation. It can be seen that the forging temperature of the module was 1106~1187 °C, the strain was 0.19~0.32, and the strain rate was 0.0032~0.0058 s^−1^. It shows that the forging process parameters were all in the first suitable processing zone.

**Figure 17:**
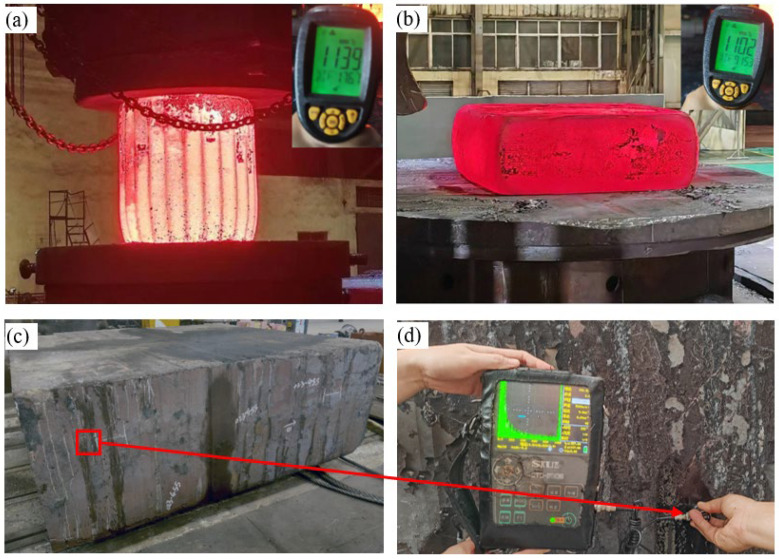
The 183 t forging of a novel Cr-Mo-V die steel: (**a**) upsetting, (**b**) forged product, (**c**) finished forging, and (**d**) ultrasonic testing.

**Figure 18:**
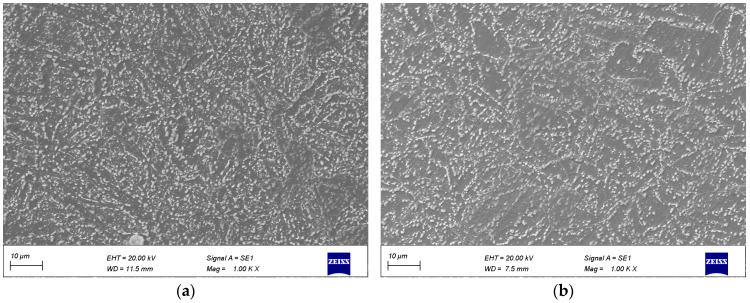
SEM images at the surface and 1/2 height of the 183 t forging. (**a**) surface; (**b**) 1/2 height.

Based on the simulation results, a novel Cr-Mo-V module was manufactured through a 1:1 forging process. The 183 t ingot was upset to H = 1800 mm using a 185 MN oil press, with a measured forging temperature of approximately 1139 °C. No longitudinal cracks occurred during this upsetting process. For each hammer used in forging, a reduction of 20% was applied at a temperature around 1102 °C, without any occurrence of longitudinal cracks, fractures, or other defects throughout the entire forging process. Figure 17c displays the finished module forging, exhibiting excellent surface quality. To observe microstructure characteristics, the surface area of the module body and the half-height test block were examined, resulting in Figure 18. The surface microstructure exhibited homogeneity, primarily consisting of granular pearlite. At half height, the organization became more uniform, with granular pearlite being dominant.

## 4. Conclusions

In this study, the thermal deformation behavior of novel Cr-Mo-V die steel at a deformation temperature of 950~1200 °C and strain rate of 0.001~5 s^−1^ was analyzed through the thermal compression test. The main conclusions were as follows:

(1) The high-temperature flow stress curve of the test steel presented two types. One had typical dynamic recovery characteristics; for example, under the conditions of a temperature of 1050 °C and a strain rate of 1 s^−1^, the stress increased rapidly with the increase in strain. When the strain reached 0.38, the stress value was 136.8 MPa, and then tended to be stable. The other had obvious dynamic recrystallization characteristics; for example, under the conditions of a temperature of 1200 °C and a strain rate of 0.01 s^−1^, when the strain reached 0.16, the maximum stress value was 37.6 MPa, and then the stable stress dropped to about 31.4 MPa.
(2) The constitutive equation of high-temperature flow stress of the tested steel was obtained: $\dot{\varepsilon }=2.78278\times 10^{20}{\left[\sinh(0.010493\sigma )\right]}^{5.87594}\times \exp(\frac{-540489.104}{RT})$. The Arrhenius model based on strain compensation was used to verify the constitutive equation, and the calculated values were in good agreement with the experimental values. The R and AARE reached 0.98902 and 3.21%, respectively. This can provide an accurate mechanical model for the forming process of the large die steel forgings of this steel.
(3) The 3D hot processing map of the novel Cr-Mo-V die steel was established based on the DMM, and the most suitable working zone was determined. The first suitable working zone was a strain of 0.05~0.35, temperature of 1100~1750 °C, and strain rate of 0.001~0.009 s^−1^. The second suitable processing zone was a strain of 0.45~0.65, temperature of 1100~1200 °C, and strain rate of 0.0024~0.33 s^−1^.
(4) The forging process of the 183 t novel Cr-Mo-V die steel forging was developed based on the 3D hot processing map, and the numerical simulation was carried out using FEM: the upsetting temperature was 1108~1170 °C, the strain was 0.47~0.55, and the strain rate was 0.0032~0.006 s^−1^, which was in the second suitable processing zone. The forging temperature of the finished product was 1106~1187 °C, the strain was 0.19~0.32, and the strain rate was 0.0032~0.0058 s^−1^, which was in the first suitable processing zone. Through 1:1 production verification, the results showed that the forging had no forging cracks and no internal defects, which verified the validity of the novel Cr-Mo-V die steel 3D hot processing map.

## Figures and Tables

**Table 1:** Composition analysis (mass fraction, %).

C	Si	Mn	S	P	Cr	Ni	Mo	V
0.56	0.20	0.83	0.0063	0.0046	1.20	1.71	1.09	0.71

**Table 2:** α, n, Q, and lnA values of the experimental steel at different strains.

Strain	α	n	Q (KJ/mol)	lnA
0.05	0.014	6.567	677.207	62.368
0.1	0.012	5.973	627.550	57.488
0.15	0.009	5.738	582.212	49.877
0.2	0.009	5.234	548.759	47.475
0.25	0.009	4.894	533.703	46.368
0.3	0.010	4.490	531.557	46.206
0.35	0.010	4.205	509.421	44.289
0.4	0.010	4.204	497.529	43.966
0.45	0.010	4.405	467.729	40.103
0.5	0.010	4.479	463.291	39.091
0.55	0.010	4.392	481.931	40.177
0.6	0.010	4.339	495.909	43.814
0.65	0.010	4.343	506.312	45.968
0.7	0.011	4.369	526.237	49.799
0.75	0.011	4.400	530.600	51.689
0.8	0.012	4.450	531.900	53.624

**Table 3:** Polynomial coefficients of α, n, Q, and lnA, and strain.

α		n		Q		lnA	
α_0_	0.01032	n_0_	9.33464	Q_0_	576.77671	A_0_	2.353 × 10^19^
α_1_	0.21499	n_1_	−100.39864	Q_1_	5312.92398	A_1_	1.574 × 10^−58^
α_2_	−3.95003	n_2_	1240.78199	Q_2_	−93,629.51026	A_2_	1.144 × 10^96^
α_3_	28.01931	n_3_	−8076.68882	Q_3_	655,200.7581	A_3_	4.336 × 10^−72^
α_4_	−102.94333	n_4_	28,566.23042	Q_4_	−2,412,886.09099	A_4_	1.747 × 10^38^
α_5_	215.29676	n_5_	−57,482.16423	Q_5_	5,036,794.41796	A_5_	5.146 × 10^−52^
α_6_	−259.29171	n_6_	65,882.95222	Q_6_	−5,983,478.55079	A_6_	4.615 × 10^98^
α_7_	167.9055	n_7_	−40,061.26807	Q_7_	3,772,194.25551	A_7_	5.718 × 10^−63^
α_8_	−45.37176	n_8_	10,027.0368	Q_8_	−980,657.87659	A_8_	2.353 × 10^19^

## Data Availability

The original contributions presented in this study are included in the article. Further inquiries can be directed to the corresponding author.
